# Stage prediction of acute kidney injury in sepsis patients using explainable machine learning approaches

**DOI:** 10.3389/fmed.2025.1667488

**Published:** 2025-10-15

**Authors:** Zhen Quan, Zheng Han, Siyao Zeng, Lianghe Wen, Jingkai Wang, Yue Li, Hongliang Wang

**Affiliations:** ^1^The Second Clinical Medical College of Harbin Medical University, Harbin, Heilongjiang Province, China; ^2^Department of Critical Care Medicine, The Second Affiliated Hospital of Harbin Medical University, Harbin, Heilongjiang Province, China

**Keywords:** acute kidney injury, MIMIC-IV database, machine learning, prediction model, sepsis

## Abstract

**Background:**

Acute kidney injury (AKI) is a prevalent and serious complication among sepsis patients, closely associated with high mortality rates and substantial disease burden. Early prediction of AKI is vital for prompt and effective intervention and improved prognosis. This research seeks to construct and assess forecasting frameworks that leverage advanced machine learning algorithms to anticipate AKI progression in high-risk sepsis patients.

**Methods:**

This study utilized the MIMIC-IV database, a large, publicly available critical care dataset containing comprehensive, de-identified electronic health records of over 70,000 ICU admissions at Beth Israel Deaconess Medical Center, to extract sepsis patient data for model training and test. Following feature selection, various machine learning algorithms were employed, including Decision Tree (DT), Efficient Neural Network (ENet), k-Nearest Neighbor (KNN), Light Gradient Boosting Machine (LightGBM), Multi-Layer Perceptron (MLP), Multinomial Mixture Model (Multinom), Random Forest (RF), and eXtreme Gradient Boosting (XGBoost). A five-fold cross-test strategy was implemented to minimize bias and assess model performance. SHapley Additive exPlanations (SHAP) was used to interpret the results.

**Results:**

A total of 6,866 critically ill sepsis patients were analyzed, of whom 5,896 developed AKI during hospitalization The RF model demonstrated superior performance, attaining an average AUC score of 0.89 on the ROC curve. SHAP analysis provided detailed insights into feature importance, including urine output, BMI, SOFA score, and maximum blood urea nitrogen, enhancing the clinical applicability of the model.

**Conclusion:**

The machine learning models developed in this study effectively predicted the stages of AKI in severely ill sepsis patients, with the Random Forest model demonstrating optimal performance. SHAP analysis offered crucial insights into the risk factors, facilitating timely and personalized interventions within a clinical setting. Additional multi-center research is essential to confirm the validity of these findings and to ultimately improve patient outcomes and quality of life.

## Introduction

Acute kidney injury associated with sepsis (SA-AKI) frequently occurs as a complication among severely ill patients. SA-AKI frequently occurs as a complication among severely ill patients (1–3). Patients with SA-AKI face substantially higher mortality rates than those without AKI or those whose AKI stems from other causes (3, 4). Although numerous therapeutic strategies have been explored, no effective clinical treatment is currently available, making early identification crucial for successful intervention (5, 6). In 2023, the 28th Acute Disease Quality Initiative (ADQI) workgroup similarly emphasized the urgent need for early identification of sepsis patients at risk of developing AKI or progressing to severe and/or persistent AKI, which is critical for timely initiation of supportive interventions, including hemodynamic optimization, fluid management, avoidance of nephrotoxic drugs, and renal replacement therapy when indicated (7). In recent years, with the advancement of machine learning (ML) models, large amounts of clinical data have been efficiently utilized, leading to numerous studies on early prediction of SA-AKI and demonstrating high diagnostic performance in related applications such as cancer and sepsis prediction (8–14). Current research mainly focuses on binary classification to predict whether AKI occurs, which presents an apparent limitation: it does not accurately classify the data for effective clinical diagnosis and treatment, nor can it differentiate the severity of AKI across individuals. Therefore, developing an ML model capable of multi-class prediction for SA-AKI Kidney Disease: Improving Global Outcomes (KDIGO) stages is crucial for better management of SA-AKI patients, as the KDIGO classification provides internationally recognized criteria for defining and staging acute kidney injury based on serum creatinine levels and urine output.

By applying the Shapley Additive exPlanations (SHAP) method, the opaque nature typical of ML models has been partially reduced. SHAP serves as a widely-used technique in machine learning to unravel the intricate relationships between features and predictive results. SHAP provides personalized insights by explaining the role of specific features in shaping model predictions, which helps clinicians understand the changing importance of features across different severities of the disease, providing more specific targets for early individualized intervention (15).

Therefore, this study aims to develop machine learning models for AKI in sepsis patients, with the dual purpose of identifying key risk factors that may enable personalized clinical intervention and achieving two specific objectives: first, to develop an ML model that best predicts the stages of SA-AKI in sepsis patients; second, to employ SHAP in interpreting the mode, visualize the risk factors, and explain the outcomes.

## Methods

### Data source

This study retrospectively analyzed data from the MIMIC-IV database (version 2.2), encompassing records from over 50,000 ICU admissions collected between 2008 and 2019 at Boston’s Beth Israel Deaconess Medical Center (16). The MIMIC-IV database is a large, publicly available critical care dataset that is continuously updated and widely used for clinical and machine learning research. It provides comprehensive and high-resolution clinical information, including patient demographics, vital signs, laboratory results, medications, procedures, and diagnostic codes from the International Classification of Diseases, Ninth and Tenth Revisions (ICD-9 and ICD-10). All data are fully de-identified in compliance with the Health Insurance Portability and Accountability Act (HIPAA), and therefore informed consent was not required.

### Participants

**Figure 1:**
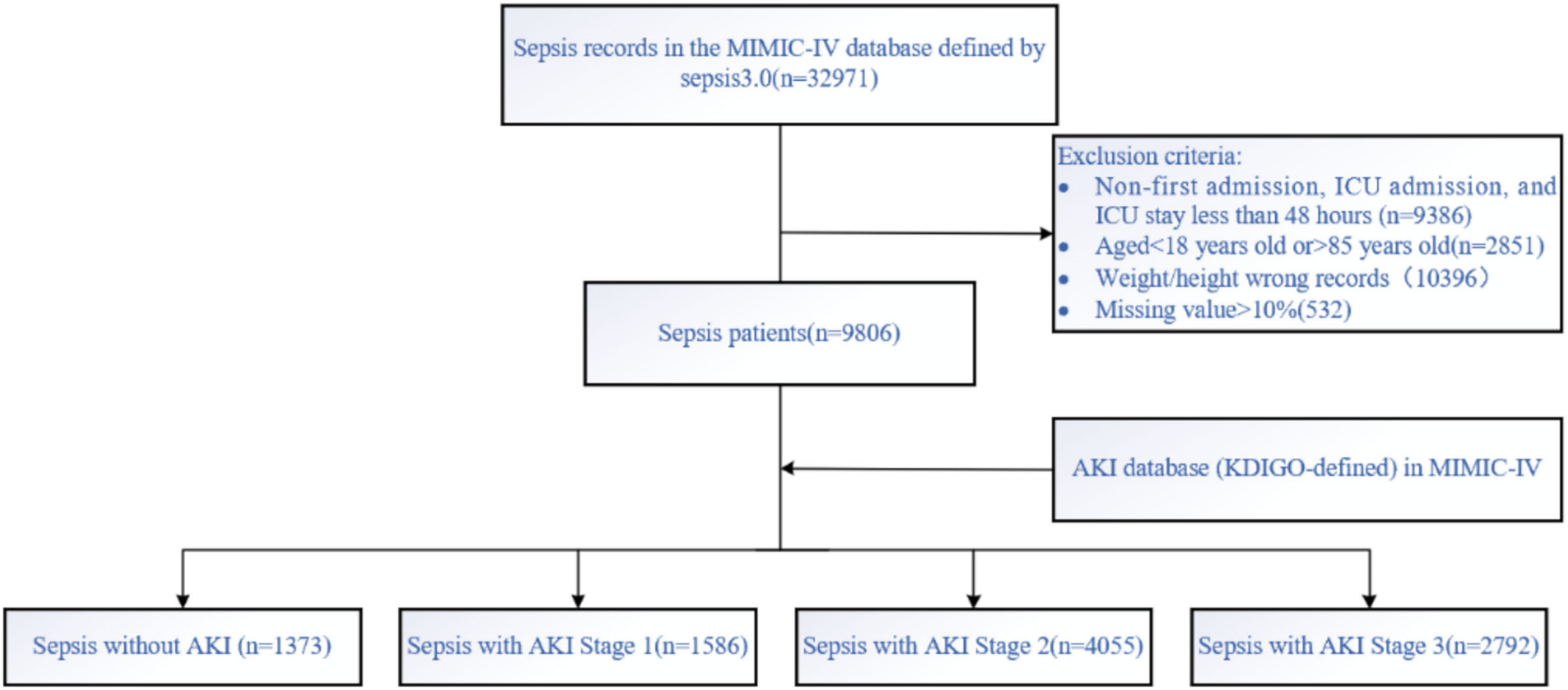
Data screening was conducted according to the Sepsis 3.0 criteria and AKI-KDIGO staging definitions, with subsequent inclusion and exclusion criteria applied.

Patients with sepsis were identified in the MIMIC-IV database according to the Sepsis-3 criteria, which define sepsis as life-threatening organ dysfunction caused by a dysregulated host response to infection. Organ dysfunction was assessed using the Sequential Organ Failure Assessment (SOFA) score, with a ≥2-point increase from baseline indicating clinically significant organ dysfunction. Patients with non-first admissions, non-first ICU stays, age <18 or >85 years, and ICU stay less than 48 h were excluded. The data were then matched with the highest KDIGO stage during the ICU stay (0, 1, 2, 3), and patients were categorized into four groups: sepsis without AKI, sepsis with AKI stage 1, 2, and 3. The KDIGO stage served as the outcome for the prediction model. The dataset was randomly split into a training set (70%) and a hold-out test set (30%) to evaluate the final model performance. No separate internal validation set was created because hyperparameter tuning and cross-validation were conducted within the training set only. The screening process is shown in Figure 1.

### Data extraction

Data extraction was performed using Navicat Premium software (version 12.0.11) and Structured Query Language (SQL). The extracted information included demographics (e.g., gender, age), comorbidities (e.g., diabetes, hypertension, pneumonia, hepatitis, heart failure), vital signs within 24 h of ICU admission (e.g., minimum/maximum systolic and diastolic blood pressure, respiratory rate, temperature, heart rate, SpO2), and laboratory indicators within 24 h of ICU admission (e.g., minimum/maximum hemoglobin, platelets, white blood cell count, anion gap, bicarbonate, blood urea nitrogen, chloride, creatinine, glucose, sodium, potassium, international normalized ratio, prothrombin time, partial thromboplastin time, SOFA score, urine output).

BMI values that were clearly implausible, resulting from data entry errors or inconsistent height and weight units, were excluded from the analysis. To reduce data bias, populations with missing values exceeding 10% were excluded, while missing values below 10% were imputed using the KNN method.

### Model development and evaluation

The dataset was imbalanced, which could affect model training and performance. Compared to binary classification tasks, multi-class imbalance problems are more complex and require more attention (17). Because the distribution of AKI stages was highly imbalanced (class 0: 1,292; class 1: 1,395; class 2: 3,587; class 3: 2,573), we applied the Synthetic Minority Oversampling Technique (SMOTE), introduced by Chawla et al. (18) to the training set to balance class sizes to approximately 4,055 samples per class before model training. A five-fold cross-validation strategy was performed within the training set to optimize hyperparameters and prevent overfitting. The final model was trained on the training set and evaluated on the independent test set, with performance assessed by average ROC–AUC and calibration metrics.

### Machine learning models

The dataset was input into seven machine learning algorithms: Decision Tree (DT), Efficient Neural Network (ENet), K-Nearest Neighbor (KNN), Light Gradient Boosting Machine (LightGBM), Multi-Layer Perceptron (MLP), Multinomial Mixture Model (Multinom), Random Forest Model (RF), and Extreme Gradient Boosting (XGBoost).

Eight machine learning models were used to predict the stages of AKI. Model evaluation metrics included Accuracy, Balanced Accuracy (Bal Accuracy), Detection Prevalence, F Measure (F Meas), Jaccard index (J index), Kappa (Kap), Matthews Correlation Coefficient (MCC), Negative Predictive Value (NPV), Positive Predictive Value (PPV), Precision, Recall, Area Under the Curve (AUC), Sensitivity (Sens), and Specificity (Spec).

For all machine learning models, key hyperparameters were tuned using grid search within reasonable ranges based on previous studies (19) and recent evidence of its effectiveness in clinical prediction contexts. Parameters were selected by 5-fold cross-validation within the training set. Final parameters were as follows:

Decision Tree: max_depth = 5

Efficient Neural Network: alpha = 1.0,l1_ratio = 0.5, max_iter = 1,000, random_state = 42

K-Nearest Neighbor: n_neighbors = 5

Light Gradient Boosting Machine: n_estimators = 200, num_leaves = 31, learning_rate = 0.1

Multi-Layer Perceptron: hidden_layer_sizes = (100, 50), activation = ‘relu’, solver = ‘adam’, learning_rate_init = 0.001

Multinomial Mixture Model: penalty = ‘l2’, C = 1.0, solver = ‘lbfgs’, multi_class = ‘multinomial’, max_iter = 1,000, random_state = 42

Random Forest Model:n_estimators = 100,random_state = 42,h_jobs = −1

Extreme Gradient Boosting: n_estimators = 200, max_depth = 6, learning_rate = 0.1

## Results

### Baseline characteristics and feature selection

Following screening and data imputation, the training set comprised a total of 6,866 patients, including 970 patients with sepsis without AKI (14.12%), 1,102 patients with SA-AKI stage 1 (16.05%), 2,839 patients with SA-AKI stage 2 (41.35%), and 1,955 patients with SA-AKI stage 3 (28.47%). Differences in characteristics among the groups are shown in Table 1. Initially, univariable analysis was conducted on these features, and those with statistical significance were subsequently included in multivariable analysis. Features with statistical significance in both univariable and multivariable analyses were adopted for model training.

**Table 1:** AKI stage 0: sepsis without AKI; hepatitis, diabetes, hypertension, pneumonia, heart failure: 0 indicates absence of the comorbidity, 1 indicates presence of the comorbidity.

AKI stage	Variable/Comorbidity	0 (*N* = 970)	1 (*N* = 1,102)	2 (*N* = 2,839)	3 (*N* = 1,955)	OR (univariable)	OR (multivariable)
Gender	Male	430 (44.3%)	364 (33%)	1,095 (38.6%)	772 (39.5%)	1.31 (1.14–1.50, *p* < 0.001)	1.50 (1.28–1.77, *p* < 0.001)
	Female	540 (55.7%)	738 (67%)	1,744 (61.4%)	1,183 (60.5%)		
Diabetes	0	662 (68.2%)	732 (66.4%)	1,821 (64.1%)	1,120 (57.3%)	1.30 (1.13–1.50, *p* < 0.001)	0.89 (0.74–1.07, *p* = 0.202)
	1	308 (31.8%)	370 (33.6%)	1,018 (35.9%)	835 (42.7%)		
Hypertension	0	393 (40.5%)	488 (44.3%)	1,076 (37.9%)	1,145 (58.6%)	0.80 (0.70–0.92, *p* = 0.002)	1.15 (0.96–1.38, *p* = 0.136)
	1	577 (59.5%)	614 (55.7%)	1,763 (62.1%)	810 (41.4%)		
Pneumonia	0	660 (68%)	710 (64.4%)	1,848 (65.1%)	1,061 (54.3%)	1.34 (1.16–1.55, *p* < 0.001)	1.75 (1.46–2.09, *p* < 0.001)
	1	310 (32%)	392 (35.6%)	991 (34.9%)	894 (45.7%)		
Heart failure	0	750 (77.3%)	734 (66.6%)	1,900 (66.9%)	1,162 (59.4%)	1.89 (1.61–2.21, *p* < 0.001)	1.58 (1.29–1.94, *p* < 0.001)
	1	220 (22.7%)	368 (33.4%)	939 (33.1%)	793 (40.6%)		
Hepatitis	0	916 (94.4%)	1,069 (97%)	2,719 (95.8%)	1,788 (91.5%)	0.97 (0.72–1.31, *p* = 0.859)	
	1	54 (5.6%)	33 (3%)	120 (4.2%)	167 (8.5%)		
Age	Mean ± SD	61.7 ± 14.6	63.8 ± 14.4	66.4 ± 12.9	65.2 ± 13.1	1.02 (1.01–1.02, *p* < 0.001)	1.01 (1.00–1.02, *p* < 0.001)
BMI	Mean ± SD	27.0 ± 5.9	28.2 ± 6.1	30.2 ± 6.9	31.6 ± 8.0	1.08 (1.07–1.09, *p* < 0.001)	1.10 (1.09–1.12, *p* < 0.001)
Hemoglobin_min	Mean ± SD	10.4 ± 2.2	9.9 ± 2.3	10.0 ± 2.2	9.6 ± 2.3	0.90 (0.87–0.93, *p* < 0.001)	0.92 (0.85–0.99, *p* = 0.028)
Hemoglobin_max	Mean ± SD	11.9 ± 2.1	11.7 ± 2.1	11.8 ± 2.0	11.3 ± 2.2	0.95 (0.92–0.98, *p* < 0.001)	1.05 (0.97–1.13, *p* = 0.193)
Platelets_min	Mean ± SD	192.5 ± 101.4	176.8 ± 89.8	177.1 ± 94.3	169.1 ± 107.2	1.00 (1.00–1.00, *p* < 0.001)	1.00 (1.00–1.00, *p* = 0.303)
Platelets_max	Mean ± SD	233.4 ± 116.2	221.4 ± 100.8	223.3 ± 106.7	218.7 ± 122.1	1.00 (1.00–1.00, *p* = 0.002)	1.00 (1.00–1.00, *p* = 0.538)
Wbc_min	Mean ± SD	10.4 ± 7.3	10.8 ± 7.8	10.8 ± 5.7	11.3 ± 7.5	1.02 (1.00–1.03, *p* = 0.014)	0.99 (0.97–1.02, *p* = 0.625)
Wbc_max	Mean ± SD	14.3 ± 10.3	15.8 ± 10.7	15.8 ± 7.7	16.6 ± 12.1	1.03 (1.02–1.04, *p* < 0.001)	1.01 (0.99–1.03, *p* = 0.349)
Aniongap_min	Mean ± SD	12.4 ± 3.1	12.3 ± 3.3	12.2 ± 3.1	14.5 ± 4.4	1.05 (1.03–1.07, *p* < 0.001)	1.05 (1.01–1.09, *p* = 0.027)
Aniongap_max	Mean ± SD	15.8 ± 4.4	15.6 ± 4.5	15.5 ± 4.3	18.9 ± 6.0	1.04 (1.02–1.05, *p* < 0.001)	0.98 (0.95–1.01, *p* = 0.268)
Bicarbonate_min	Mean ± SD	22.0 ± 4.5	21.8 ± 4.0	21.8 ± 4.2	19.7 ± 5.4	0.96 (0.95–0.97, *p* < 0.001)	0.95 (0.91–0.99, *p* = 0.023)
Bicarbonate_max	Mean ± SD	24.7 ± 4.0	24.6 ± 3.7	24.6 ± 3.9	23.5 ± 4.8	0.97 (0.96–0.99, *p* < 0.001)	1.06 (1.02–1.11, *p* = 0.005)
Bun_min	Mean ± SD	19.4 ± 17.7	22.0 ± 18.8	20.7 ± 14.6	34.0 ± 24.8	1.02 (1.02–1.03, *p* < 0.001)	1.02 (1.00–1.04, *p* = 0.051)
Bun_max	Mean ± SD	24.0 ± 21.4	26.4 ± 22.2	24.7 ± 17.0	41.5 ± 28.7	1.02 (1.01–1.02, *p* < 0.001)	0.97 (0.96–0.99, *p* = 0.002)
Chloride_min	Mean ± SD	101.9 ± 6.2	102.6 ± 6.2	102.8 ± 5.9	100.7 ± 7.1	1.00 (0.99–1.01, *p* = 0.481)	
Chloride_max	Mean ± SD	106.4 ± 6.3	107.2 ± 6.5	107.0 ± 5.9	105.2 ± 7.0	1.00 (0.99–1.01, *p* = 0.837)	
Creatinine_min	Mean ± SD	1.0 ± 0.9	1.2 ± 1.2	1.0 ± 0.7	2.0 ± 1.9	1.62 (1.45–1.81, *p* < 0.001)	0.98 (0.70–1.35, *p* = 0.882)
Creatinine_max	Mean ± SD	1.2 ± 1.3	1.4 ± 1.5	1.2 ± 0.8	2.6 ± 2.4	1.45 (1.34–1.58, *p* < 0.001)	1.13 (0.87–1.47, *p* = 0.341)
Glucose_min	Mean ± SD	116.9 ± 32.7	119.8 ± 37.3	123.2 ± 39.4	122.1 ± 46.2	1.00 (1.00–1.01, *p* < 0.001)	1.00 (1.00–1.01, *p* = 0.007)
Glucose_max	Mean ± SD	164.9 ± 102.7	165.2 ± 89.9	168.8 ± 89.0	193.2 ± 114.5	1.00 (1.00–1.00, *p* < 0.001)	1.00 (1.00–1.00, *p* = 0.828)
Sodium_min	Mean ± SD	136.8 ± 4.9	136.8 ± 4.6	137.0 ± 4.8	136.2 ± 5.7	1.00 (0.98–1.01, *p* = 0.487)	
Sodium_max	Mean ± SD	140.2 ± 5.0	140.2 ± 4.8	140.1 ± 4.6	139.8 ± 5.5	0.99 (0.98–1.01, *p* = 0.316)	
Potassium_min	Mean ± SD	3.8 ± 0.5	3.9 ± 0.6	3.9 ± 0.5	4.0 ± 0.7	1.55 (1.38–1.75, *p* < 0.001)	0.87 (0.73–1.05, *p* = 0.155)
Potassium_max	Mean ± SD	4.5 ± 0.7	4.6 ± 0.8	4.6 ± 0.7	4.9 ± 1.0	1.58 (1.43–1.75, *p* < 0.001)	1.22 (1.06–1.39, *p* = 0.004)
INR_min	Mean ± SD	1.3 ± 0.4	1.3 ± 0.7	1.3 ± 0.4	1.5 ± 0.7	1.68 (1.38–2.05, *p* < 0.001)	0.48 (0.20–1.14, *p* = 0.098)
INR_max	Mean ± SD	1.4 ± 0.7	1.6 ± 1.2	1.5 ± 1.0	1.9 ± 1.5	1.59 (1.39–1.83, *p* < 0.001)	1.28 (0.92–1.77, *p* = 0.139)
Pt_min	Mean ± SD	13.8 ± 4.4	14.3 ± 5.8	14.1 ± 4.1	16.2 ± 7.6	1.05 (1.03–1.08, *p* < 0.001)	1.07 (0.98–1.17, *p* = 0.119)
Pt_max	Mean ± SD	15.5 ± 8.1	17.3 ± 11.7	16.7 ± 9.9	20.7 ± 15.5	1.05 (1.03–1.06, *p* < 0.001)	0.99 (0.96–1.02, *p* = 0.474)
Ptt_min	Mean ± SD	30.6 ± 11.3	30.6 ± 9.8	30.8 ± 11.0	33.1 ± 11.6	1.01 (1.00–1.02, *p* = 0.013)	1.00 (0.99–1.00, *p* = 0.325)
Ptt_max	Mean ± SD	41.0 ± 27.9	43.1 ± 27.1	44.9 ± 30.4	51.6 ± 35.4	1.01 (1.00–1.01, *p* < 0.001)	1.00 (1.00–1.01, *p* = 0.035)
Sofa	Mean ± SD	4.1 ± 3.0	5.2 ± 3.1	5.5 ± 3.2	8.2 ± 4.0	1.23 (1.20–1.26, *p* < 0.001)	1.15 (1.12–1.19, *p* < 0.001)
Urine output	Mean ± SD	2,535.9 ± 1,376.0	2,408.2 ± 1,304.9	1,814.0 ± 1,006.3	1,133.2 ± 1,054.7	1.00 (1.00–1.00, *p* < 0.001)	1.00 (1.00–1.00, *p* < 0.001)
Heartrate_min	Mean ± SD	71.2 ± 15.5	69.9 ± 14.0	70.3 ± 14.9	72.4 ± 16.8	1.00 (0.99–1.00, *p* = 0.673)	
Heartrate_max	Mean ± SD	105.1 ± 20.5	104.0 ± 20.5	104.1 ± 19.9	108.1 ± 23.4	1.00 (1.00–1.00, *p* = 0.707)	
Sbp_min	Mean ± SD	93.3 ± 17.5	89.0 ± 15.4	88.6 ± 15.8	86.2 ± 17.1	0.98 (0.98–0.98, *p* < 0.001)	1.00 (0.99–1.00, *p* = 0.277)
Sbp_max	Mean ± SD	148.9 ± 22.7	147.9 ± 22.5	149.0 ± 23.2	147.4 ± 24.9	1.00 (1.00–1.00, *p* = 0.452)	
Dbp_min	Mean ± SD	48.5 ± 11.3	45.8 ± 9.8	45.5 ± 10.0	44.0 ± 11.3	0.97 (0.96–0.98, *p* < 0.001)	1.00 (0.99–1.00, *p* = 0.338)
Dbp_max	Mean ± SD	89.0 ± 18.8	84.4 ± 17.0	85.2 ± 19.2	86.6 ± 20.6	0.99 (0.99–0.99, *p* < 0.001)	1.00 (0.99–1.00, *p* = 0.579)
Resprate_min	Mean ± SD	12.4 ± 3.5	12.1 ± 3.6	12.0 ± 3.7	12.6 ± 4.2	0.99 (0.97–1.00, *p* = 0.154)	
Resprate_max	Mean ± SD	28.1 ± 6.3	27.8 ± 6.2	28.1 ± 6.7	29.1 ± 6.6	1.01 (1.00–1.02, *p* = 0.218)	
Temperature_min	Mean ± SD	36.4 ± 0.6	36.2 ± 0.9	36.2 ± 0.8	36.2 ± 0.9	0.69 (0.62–0.77, *p* < 0.001)	0.73 (0.65–0.83, *p* < 0.001)
Temperature_max	Mean ± SD	37.5 ± 0.7	37.5 ± 0.8	37.5 ± 0.8	37.5 ± 0.9	0.97 (0.89–1.05, *p* = 0.446)	
Spo2_min	Mean ± SD	92.5 ± 5.4	92.3 ± 5.8	92.1 ± 5.3	90.6 ± 8.5	0.97 (0.96–0.99, *p* < 0.001)	1.00 (0.99–1.02, *p* = 0.933)
Spo2_max	Mean ± SD	99.6 ± 1.0	99.7 ± 0.9	99.7 ± 0.9	99.6 ± 1.0	1.07 (1.01–1.15, *p* = 0.029)	1.05 (0.97–1.14, *p* = 0.238)

### Model performance

**Figure 2:**
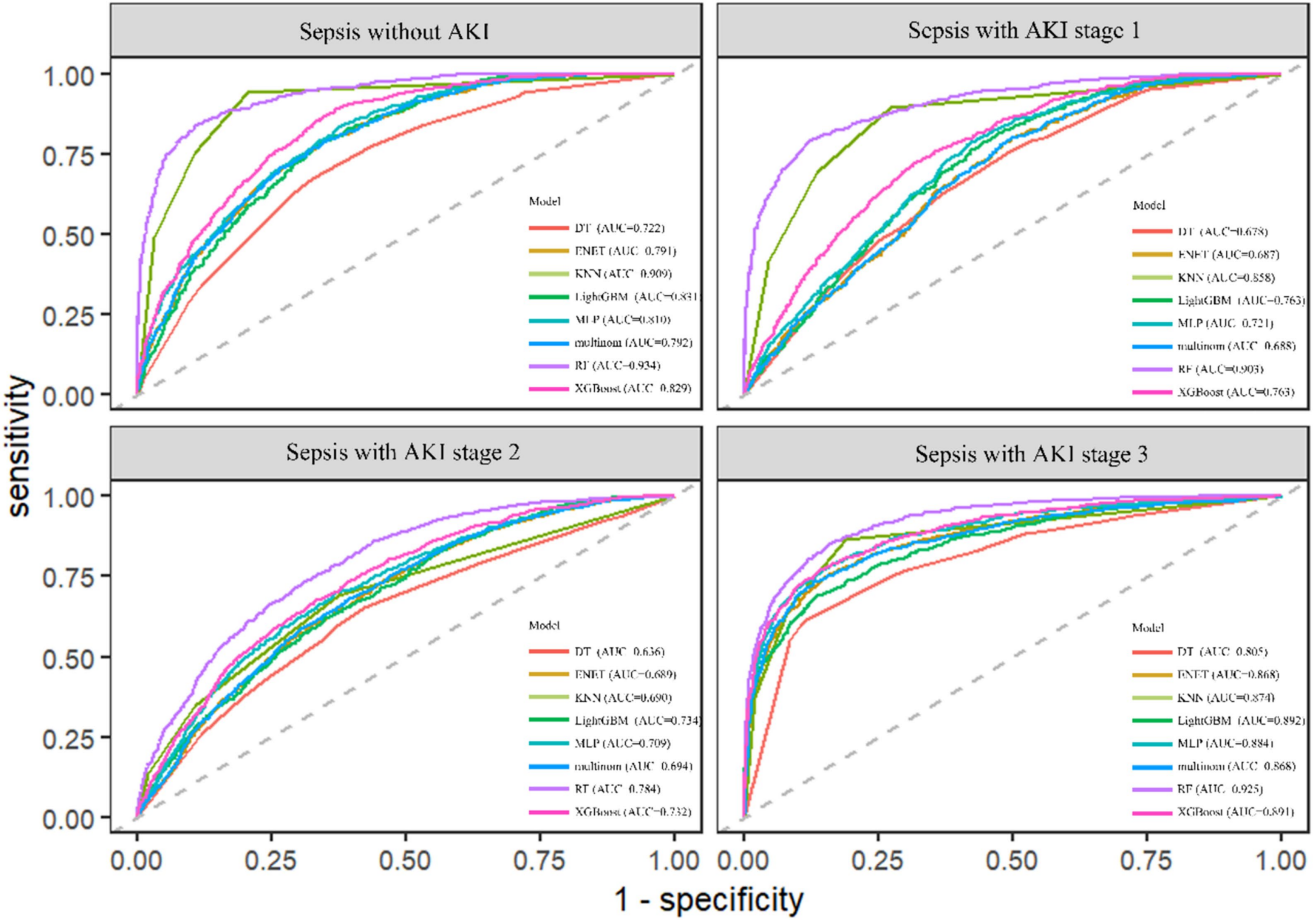
ROC–AUC of eight machine learning models for the four AKI stages.

**Figure 3:**
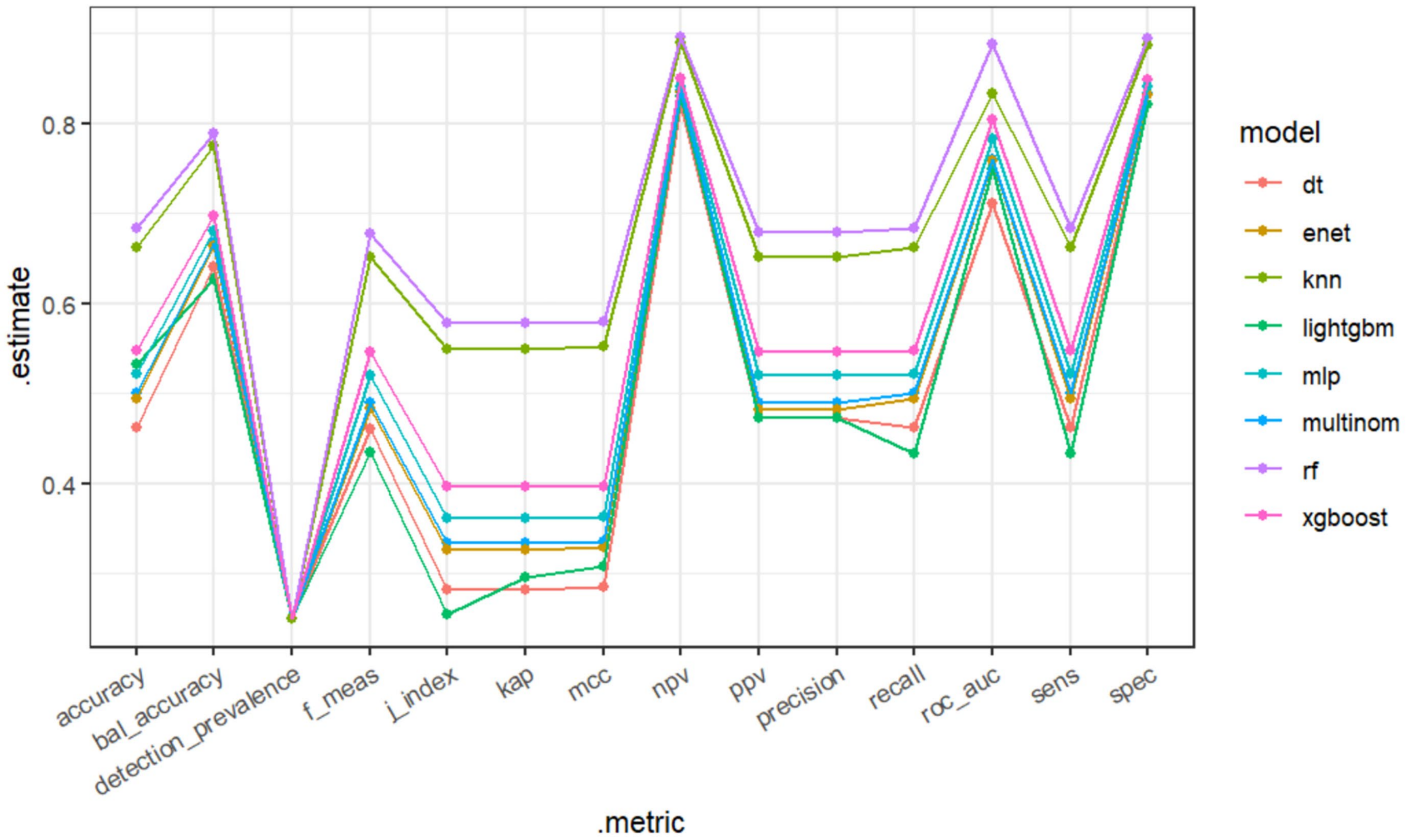
Comparison of eight machine learning models based on a Line graph.

The RF model demonstrated the highest performance, achieving an average macro-AUC of 0.888 across all AKI stages during five-fold cross-validation (Supplementary Figure 1). In the independent test set, the ROC–AUC values for each class were: sepsis without AKI, 0.934; SA-AKI stage 1, 0.903; SA-AKI stage 2, 0.784; and SA-AKI stage 3, 0.925 (Figure 2). The ROC–AUC values of the other models were as follows: Multinomial Mixture Model (Multinom), 0.760; Efficient Neural Network (ENet), 0.759; Decision Tree (DT), 0.710; XGBoost, 0.804; Multi-Layer Perceptron (MLP), 0.782; LightGBM, 0.750; and k-Nearest Neighbor (KNN), 0.833 (Table 2, Figure 3). Comparison between training and test sets showed similar AUC distributions (Supplementary Figure 2, Table 1), highlighting the validity of the RF model. These results indicate that the RF model not only outperforms other algorithms but also maintains consistent discriminative ability across all AKI stages.

**Table 2:** Evaluation metrics values for the performance of eight machine learning models.

Model	KNN	LightGBM	MLP	XGBoost	RF	DT	ENet	Multinom
Accuracy	0.661463	0.532497	0.521569	0.546837	0.683238	0.46138	0.494864	0.5
Kap	0.548617	0.296284	0.362093	0.395782	0.57765	0.281841	0.326486	0.333333
sens	0.661463	0.43296	0.521569	0.546837	0.683238	0.46138	0.494864	0.5
spec	0.887154	0.821365	0.840523	0.848946	0.894413	0.82046	0.831622	0.833333
PPV	0.651698	0.472942	0.520259	0.54676	0.678263	0.472806	0.481882	0.488936
NPV	0.889759	0.830155	0.841067	0.849359	0.896174	0.820703	0.834332	0.835678
MCC	0.551503	0.308416	0.36276	0.396415	0.579552	0.28468	0.328856	0.33538
J index	0.548617	0.254325	0.362093	0.395782	0.57765	0.281841	0.326486	0.333333
Bal accuracy	0.774308	0.627162	0.681046	0.697891	0.788825	0.64092	0.663243	0.666667
Detection prevalence	0.25	0.25	0.25	0.25	0.25	0.25	0.25	0.25
Precision	0.651698	0.472942	0.520259	0.54676	0.678263	0.472806	0.481882	0.488936
Recall	0.661463	0.43296	0.521569	0.546837	0.683238	0.46138	0.494864	0.5
F meas	0.651786	0.433932	0.519634	0.545571	0.677663	0.460908	0.483394	0.490186
ROC–AUC	0.832936	0.749651	0.782232	0.804209	0.888434	0.710227	0.758831	0.760082

### Interpretability analysis

**Figure 4:**
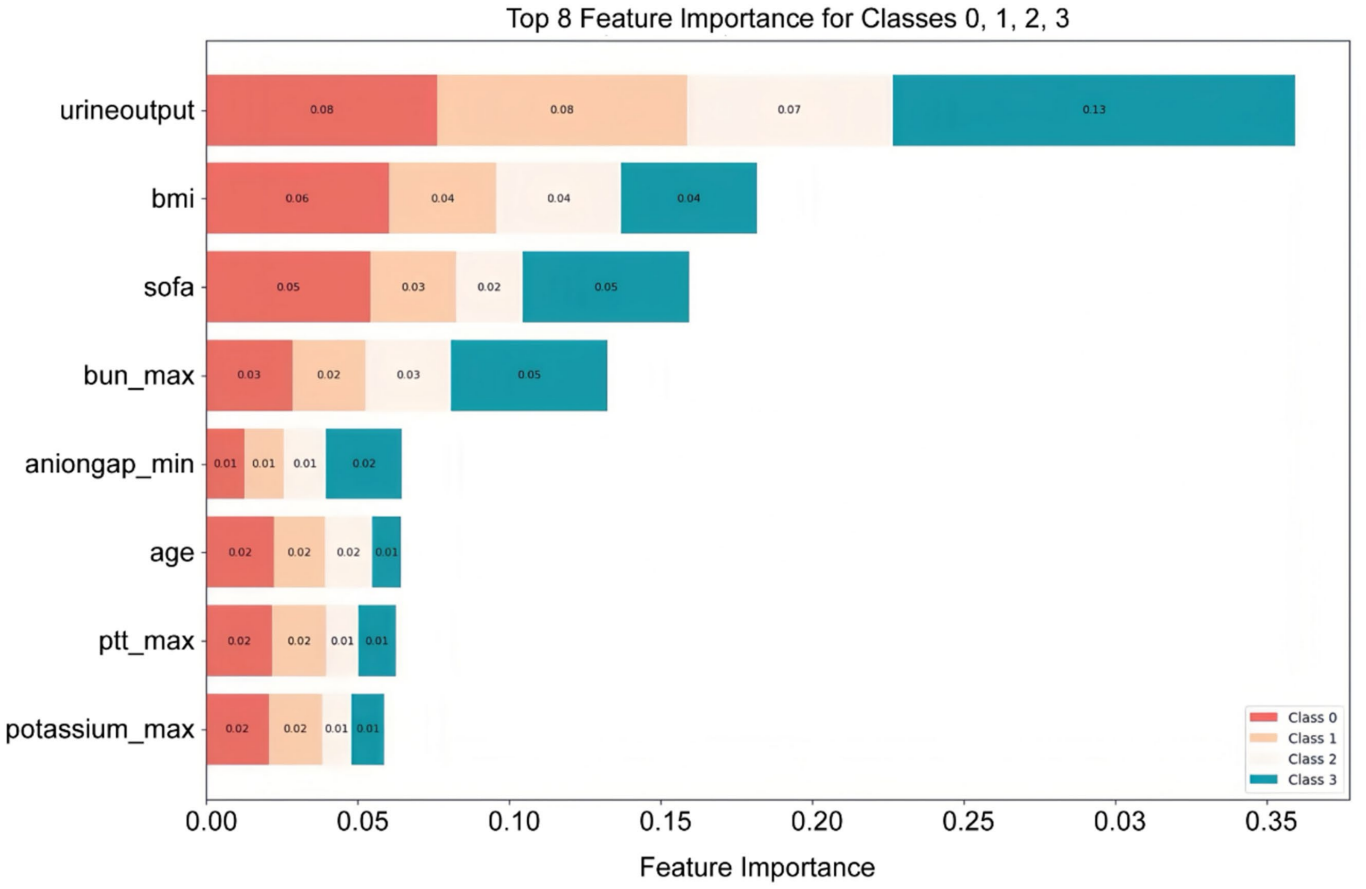
Top eight important features ranked by SHAP values: The *X*-axis represents the importance of the features, while the *Y*-axis shows the different features; Class 0, 1, 2, and 3 represent sepsis without AKI, and sepsis with AKI stages 1, 2, and 3, respectively.

**Figure 5:**
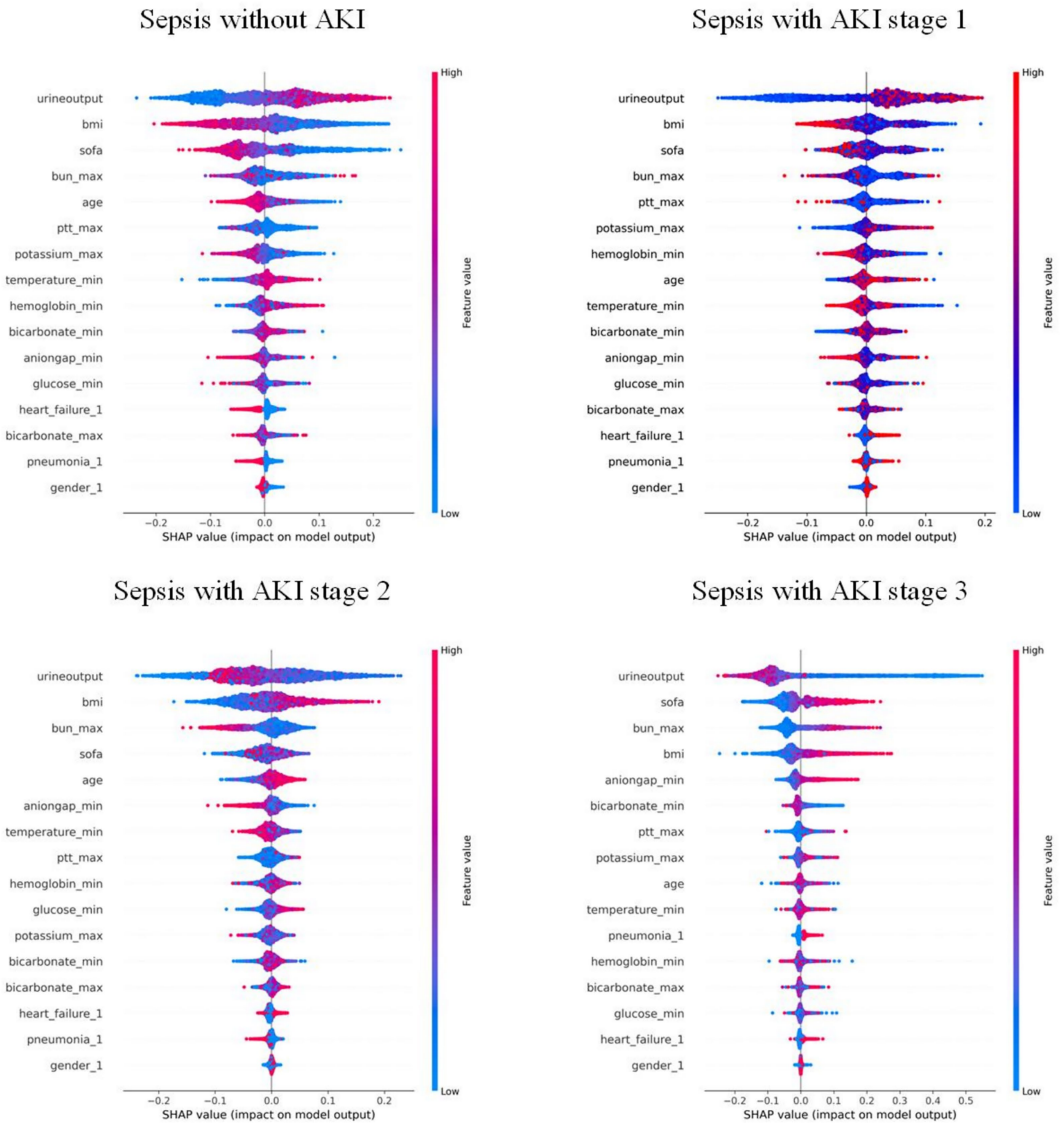
Bee swarm plot of the RF model: each point represents the data of a patient within the corresponding class. Red indicates relatively high values, and blue indicates relatively low values. The *X*-axis represents the magnitude of SHAP values, while the *Y*-axis shows features ranked by importance from top to bottom.

Features were ranked by SHAP values in descending order, which helps analyze the occurrence of AKI and display the importance of different predictive variables across groups. Figure 4 shows the top eight important features, while Figure 5 presents the SHAP bee swarm plot for four groups Each patient’s feature is depicted as a dot, with colors reflecting attribution values: red for higher values and blue for lower values. Urine output, BMI, SOFA score, and maximum blood urea nitrogen were the most important factors across groups. The importance of different features varied among groups; for example, the importance of SOFA score and minimum anion gap was positively correlated with AKI stage severity.

**Figure 6:**
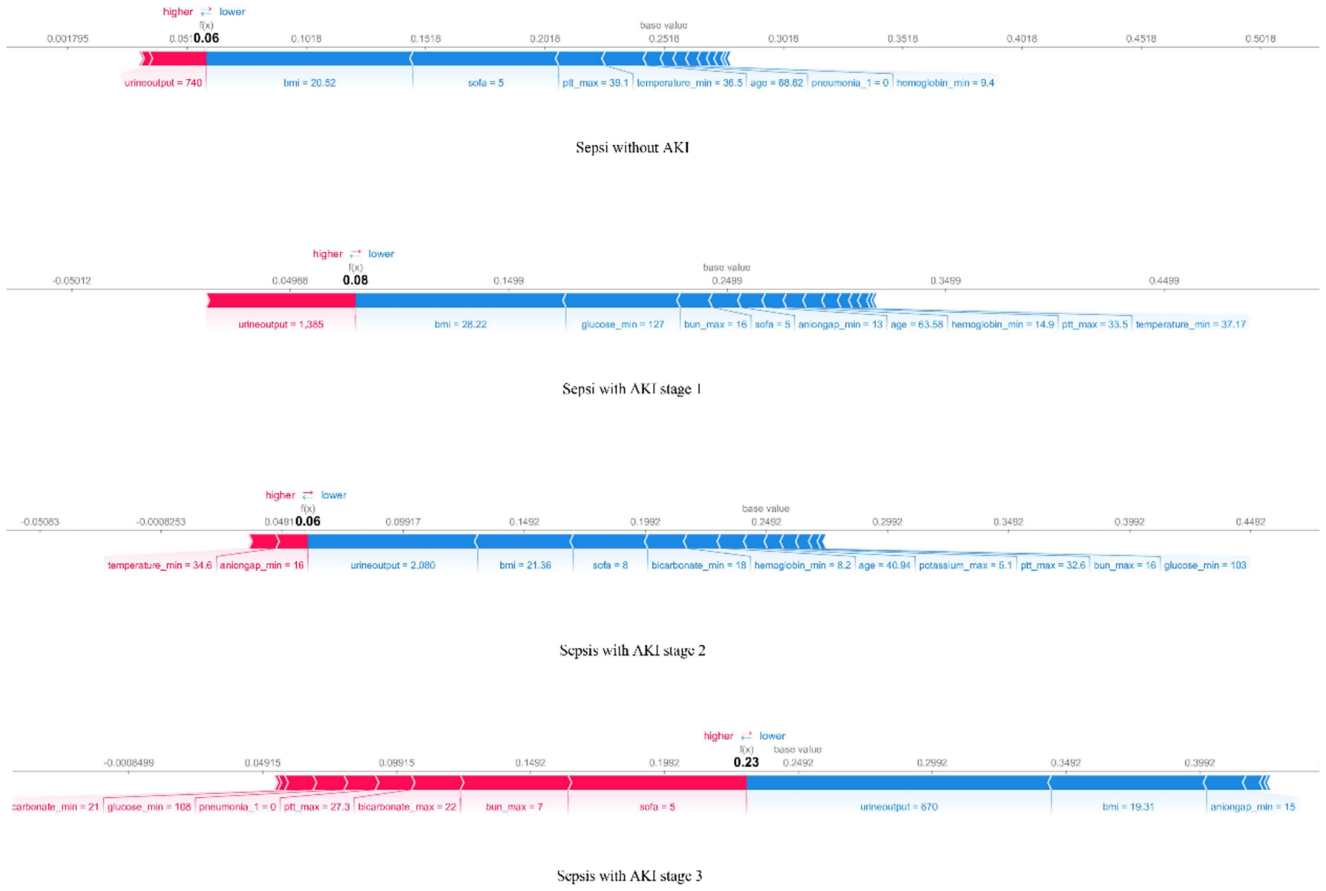
The force plot of the RF model visualizes the result for a randomly selected patient from the four groups. The base value represents the average predicted outcome, with feature values and names listed at the bottom of the plot. Features are sorted from the center outward based on their impact on the prediction.

The SHAP force plot (Figure 6) helps understand local interpretability (i.e., individual patients) by showing how features contribute to the prediction for a particular patient. The force plot displays whether a feature promotes or inhibits the prediction outcome and shows its relative strength, providing explicit guidance for clinical diagnosis and treatment.

The SHAP dependence plot (Supplementary Figures 3–5) displays the interaction effects between features, showing how two primary features influence each other.

## Discussion

This study involved screening data from the MIMIC-IV database to examine multiple indicators of sepsis patients within the first 24 h of ICU admission and their associations with the occurrence and progression of AKI. Currently, dozens of studies on sepsis use this database to construct ML models, making our use of the same data reasonable and feasible (20–22).

We used univariable and multivariable analyses to identify 16 early clinical parameters for developing and validating the prediction model. The results showed that the RF model exhibited better discrimination and calibration capabilities than other ML algorithms. Compared with traditional logistic regression or simple scoring systems, our multi-class RF model integrates multiple routinely collected clinical indicators and provides more accurate and granular risk stratification, which enhances its potential for bedside application. To investigate how these features influence RF algorithm decisions, we used SHAP to interpret predictions. The SHAP bee swarm plots illustrated the importance of features across the different groups, while the dependence plots demonstrated feature relationships and their effect on model measurement. Additionally, SHAP force plots and waterfall plots illustrated how the model locally explained the relationship between feature and sepsis prediction.

SA-AKI is a sepsis complication with high mortality. Although several novel biomarkers for detecting kidney injury and predicting AKI development—such as NGAL, KIM-1, cystatin C, and IL-18—have been discovered, they are still insufficiently sensitive for early detection, which makes the exploration of early prediction of SA-AKI irreplaceable (5). Our approach leverages only standard clinical and laboratory data available within 24 h of ICU admission, avoiding the need for costly or time-consuming biomarker testing, and thus increasing its feasibility in routine critical care settings.

With the development of artificial intelligence, ML models have become increasingly important tools in medical research. ML automatically learns patterns and features from large datasets and generates prediction and decision models to make predictions for new data. Previous studies have predominantly used binary classification to predict whether SA-AKI occurs, but the severity classification of AKI is crucial for treatment and prognosis. Studies have shown that the higher the AKI stage, the greater the likelihood of requiring renal replacement therapy, and the higher the mortality rate (23). Therefore, this study adopted a multi-class classification approach to predict AKI KDIGO stages, which aligns better with treatment guideline variations for patients at different KDIGO stages and has greater clinical application potential. By simultaneously predicting all KDIGO stages, our model provides clinicians with a more refined tool for individualized risk assessment and early intervention planning.

An important concern in ML is the black-box issue, where early studies lacked explanations of ML models—how input variables affect model results, and to what extent are often unknown. This is one of the major barriers to clinical application. This study used SHAP to visualize and interpret the multi-class results, allowing us to see how features impact each stage of the model. Another advantage of SHAP is comparing changes in feature importance across different AKI stages, which offers a deeper understanding of how feature importance changes with disease severity and helps guide targeted treatment. For example, for a patient predicted to have a high risk of stage 2–3 AKI, SHAP analysis identified low urine output, elevated BMI, high SOFA score, and increased maximum BUN as the top contributing factors. This explanation helps clinicians understand why the model predicts high risk and facilitates targeted interventions, such as closer monitoring of renal function or adjustment of fluid and medication management. This combination of multi-class modeling and interpretable AI not only improves predictive performance but also enhances clinical trust and facilitates translation into practice.

Recent studies indicate that a high BMI is associated with the early occurrence of SA-AKI and correlates with its severity, which was also confirmed by our model. Further, by introducing SHAP, the impact of high BMI on SA-AKI occurrence was quantified and visualized (24). Multivariate regression analyses have identified SOFA score as an independent risk factor for persistent severe SA-AKI, which is consistent with the predictions of our model (25). In addition, previous studies have determined 12 risk factors associated with early SA-AKI development, including age, BMI, and urine output—key features also captured by our model (26). Since SOFA score was introduced to define sepsis, numerous studies have either modified it or combined it with other biomarkers for prediction (27, 28). By integrating ML with large and complex datasets, our model demonstrated that SOFA score is one of the important influencing factors (Figure 5). SOFA score ranked second in importance in the KDIGO stage 3 model, while ranking lower in the other three models, suggesting that its accuracy in predicting patients with different severity levels should be considered when using the SOFA score, thus underscoring the importance of a multi-class model for predicting SA-AKI.

Nonetheless, this study has certain limitations. The MIMIC-IV database originates from a single U. S. center, which may limit generalizability to other regions or populations, and the use of imputation for missing data could introduce bias. Additionally, the model has not been externally validated, which may affect its robustness. We also only used the minimum and maximum values within the first 24 h, potentially overlooking important temporal dynamics. Future work will focus on external validation in multi-center prospective cohorts across different regions and populations, exploration of novel biomarkers, incorporation of continuous time-series data (e.g., dynamic trends of creatinine and urine output) to capture temporal patterns of disease progression, and assessment of the model in real ICU settings. These improvements aim to enhance predictive accuracy, clinical applicability, and the robustness of our findings, while helping identify optimal time-points for stage-specific clinical interventions.

## Conclusion

This research effectively established robust machine learning models for predicting stages of AKI in severely ill sepsis patients, with the RF model exhibiting optimal performance. Through the application of SHAP analysis, critical risk factors such as urine output, body mass index, SOFA score, and peak blood urea nitrogen were identified, highlighting the potential for personalized risk assessment. These results lay the groundwork for early interventions, supporting improved management and survival outcomes in sepsis patients.

## Data Availability

The original contributions presented in the study are included in the article/Supplementary material, further inquiries can be directed to the corresponding author.

## Glossary

AKI: Acute kidney injury
DT: Decision Tree
Enet: efficient Neural Network
KNN: K-Nearest Neighbor
LightGBM: Light Gradient Boosting Machine
MLP: Multi-Layer Perceptron
Multinom: Multinomial Mixture Model
RF: Random Forest
XGBoost: eXtreme Gradient Boosting
SA-AKI: Acute kidney injury associated with sepsis
ADQI: Acute Disease Quality Initiative
ML: machine learning
SHAP: Shapley Additive exPlanations
SQL: Structured Query Language
SOFA: Sequential Organ Failure Assessment
SMOTE: Synthetic Minority Oversampling Technique
Bal Accuracy: Balanced Accuracy
F Meas: F Measure
J index: Jaccard index
Kap: Kappa
MCC: Matthews Correlation Coefficient
NPV: Negative Predictive Value
PPV: Positive Predictive Value
AUC: Area Under the Curve
Sens: Sensitivity
Spec: Specificity
