# Supplementary material for: Stage prediction of acute kidney injury in sepsis patients using explainable machine learning approaches
Source: Front Med (Lausanne). 2025 Oct 15;12:1667488. doi: 10.3389/fmed.2025.1667488 (PMC12568512; doi:10.3389/fmed.2025.1667488)
Supplement: Supplementary file 1 [file Data_Sheet_1.docx]

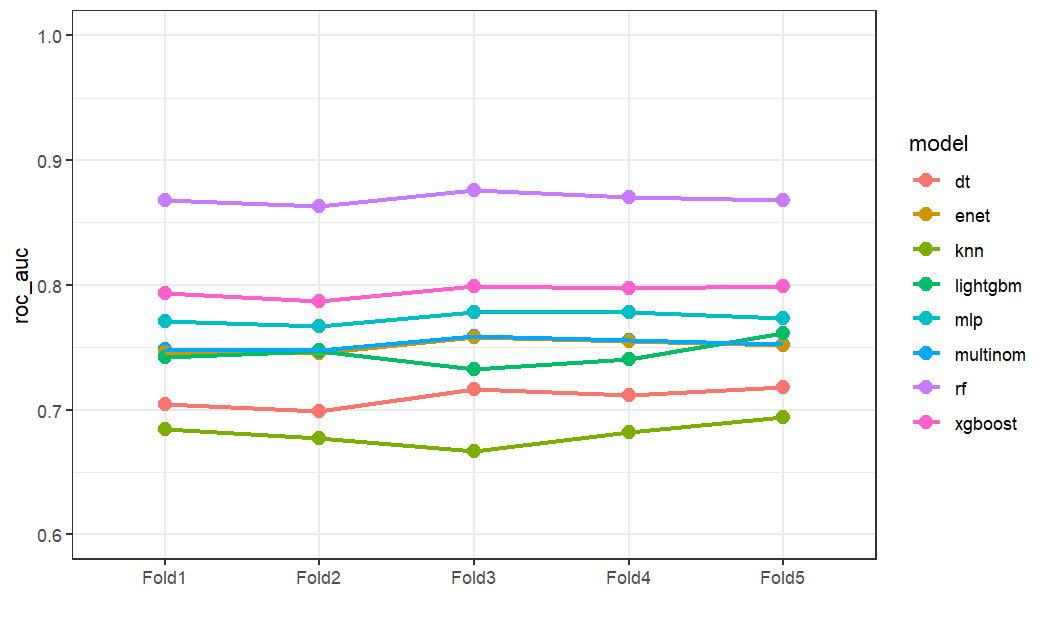
 **Supplementary Fig. 1** five-fold cross-validation


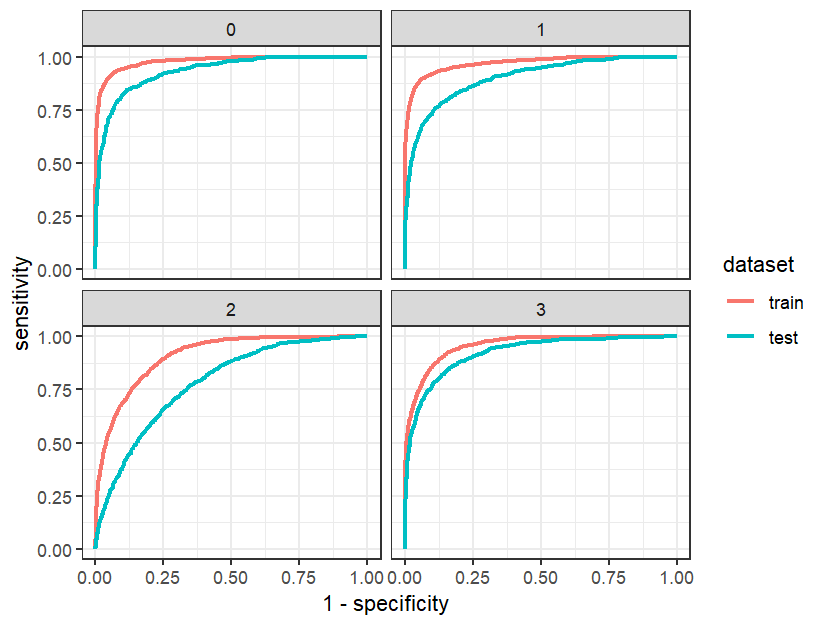
 **Supplementary Fig. 2** Comparison of ROC-AUC between training and test sets for each classification of the RF model.

**Supplementary Fig. 3-5** dependence plot displays the interaction effects between features, showing how two primary features influence each other.
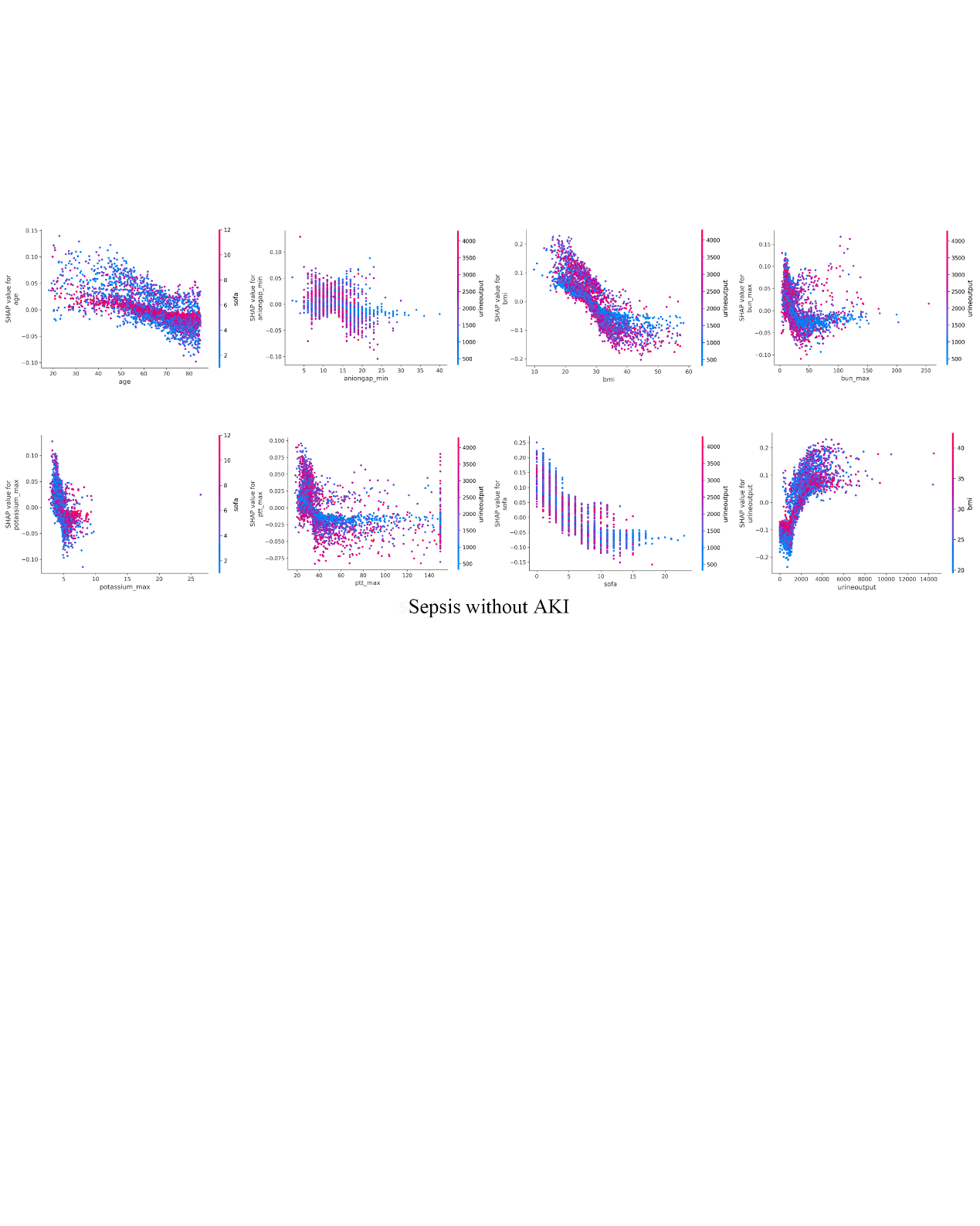


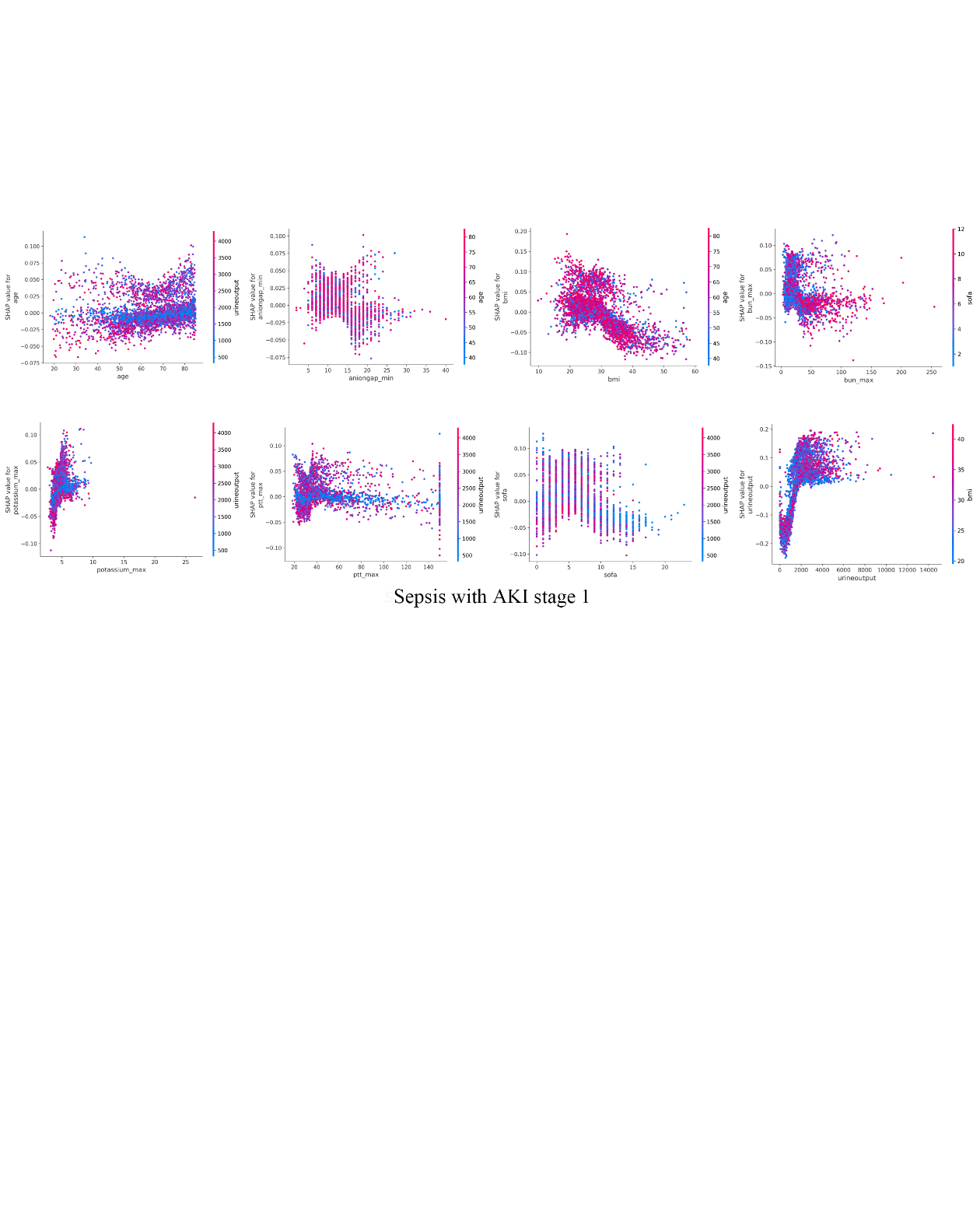


**
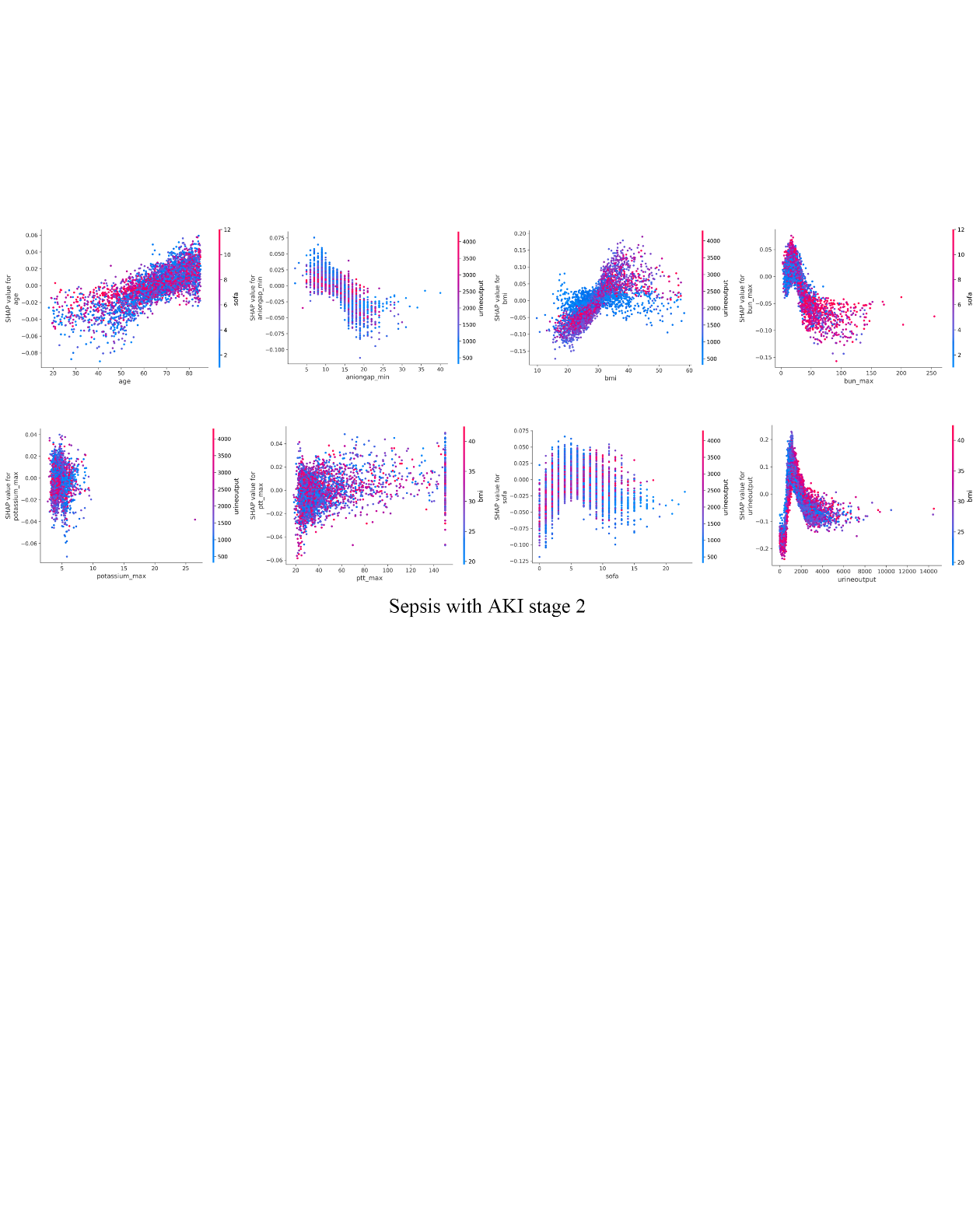
**

**
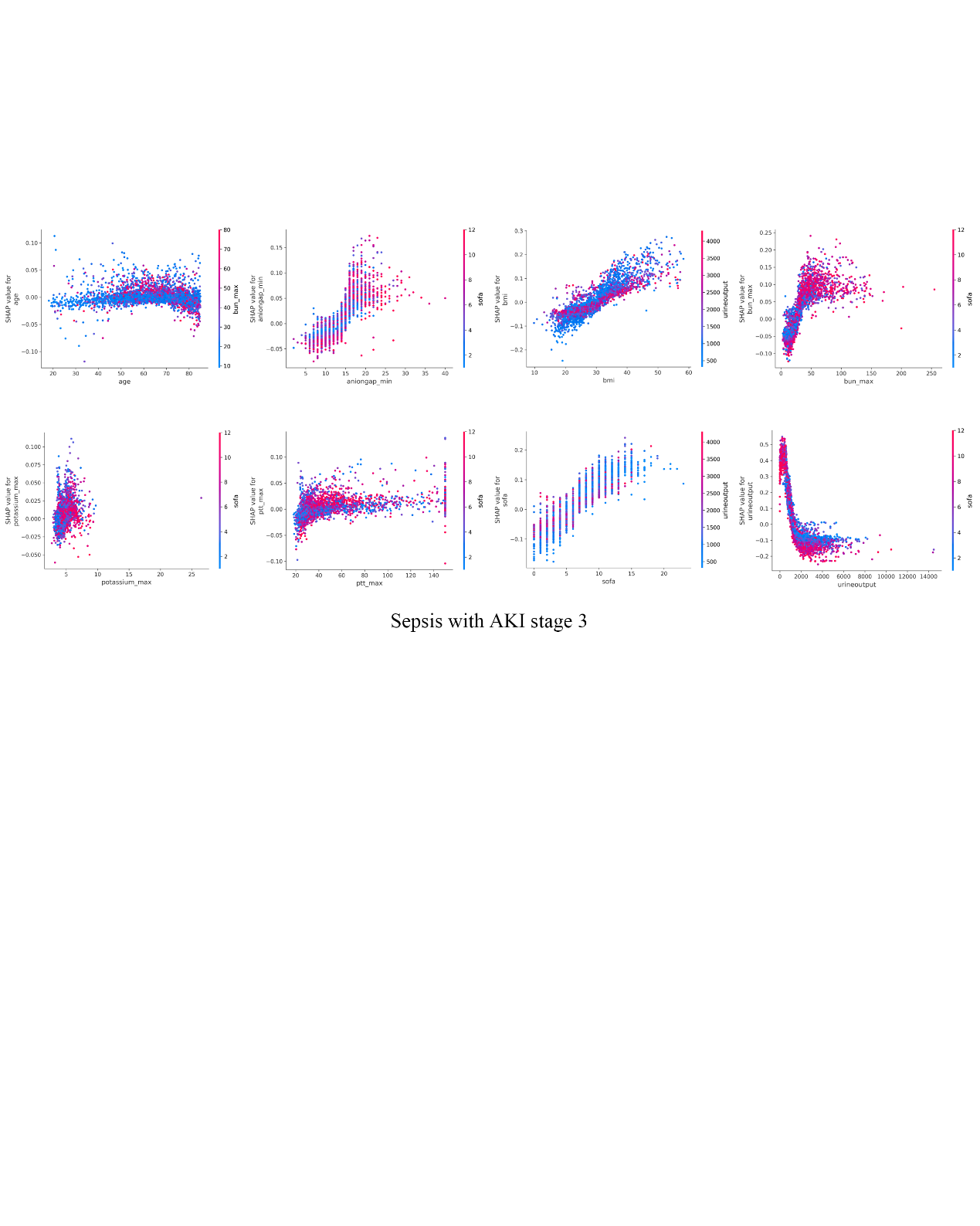
**

| Model | Class | Train | Test |
| --- | --- | --- | --- |
| RF | 0 | 0.979 | 0.934 |
|  | 1 | 0.970 | 0.903 |
|  | 2 | 0.910 | 0.784 |
|  | 3 | 0.958 | 0.925 |
| XGboost | 0 | 0.846 | 0.829 |
|  | 1 | 0.790 | 0.763 |
|  | 2 | 0.765 | 0.732 |
|  | 3 | 0.897 | 0.891 |
| MLP | 0 | 0.812 | 0.810 |
|  | 1 | 0.742 | 0.721 |
|  | 2 | 0.723 | 0.709 |
|  | 3 | 0.890 | 0.884 |
| Multinom | 0 | 0.783 | 0.792 |
|  | 1 | 0.692 | 0.688 |
|  | 2 | 0.690 | 0.694 |
|  | 3 | 0.858 | 0.868 |
| LightGBM | 0 | 0.847 | 0.831 |
|  | 1 | 0.794 | 0.763 |
|  | 2 | 0.772 | 0.734 |
|  | 3 | 0.901 | 0.892 |
| KNN | 0 | 0.987 | 0.909 |
|  | 1 | 0.980 | 0.858 |
|  | 2 | 0.940 | 0.690 |
|  | 3 | 0.982 | 0.874 |
| Enet | 0 | 0.783 | 0.791 |
|  | 1 | 0.690 | 0.687 |
|  | 2 | 0.686 | 0.689 |
|  | 3 | 0.857 | 0.868 |
| Dt | 0 | 0.727 | 0.722 |
|  | 1 | 0.683 | 0.678 |
|  | 2 | 0.649 | 0.636 |
|  | 3 | 0.814 | 0.805 |

**Supplementary Table 1.** Comparison of ROC-AUC between training and test sets for the eight models.
